# Salivary Cortisol Levels Are Associated with Craving and Cognitive Performance in Cocaine-Abstinent Subjects: A Pilot Study

**DOI:** 10.3390/brainsci10100682

**Published:** 2020-09-27

**Authors:** Patricia Sampedro-Piquero, Selene Vicario, Aroha Pérez-Rivas, César Venero, Shishir Baliyan, Luis Javier Santín

**Affiliations:** 1 Departamento de Psicobiología y Metodología de las Ciencias del Comportamiento, Facultad de Psicología, Campus de Teatinos S/N, Universidad de Málaga, 29071 Málaga, Spain; selene_malaga_5@hotmail.com (S.V.); arohaperez21@gmail.com (A.P.-R.); 2 Instituto de Investigación Biomédica de Málaga (IBIMA), 29010 Málaga, Spain; 3 Departamento de Psicobiología, Facultad de Psicología, UNED, 28040 Madrid, Spain; cvenero@psi.uned.es (C.V.); shishirbaliyan@gmail.com (S.B.)

**Keywords:** addiction, cocaine, cognition, cortisol, craving, hypothalamic-pituitary-adrenal (HPA), memory

## Abstract

Cortisol is a glucocorticoid hormone secreted by the adrenal cortex upon the activation of the hypothalamic-pituitary-adrenal (HPA) axis. Assessment of cortisol in saliva has emerged as a reliable way of evaluating HPA function. We examined the relationships between salivary cortisol levels with both craving and cognitive performance, as a possible biomarker of cocaine addiction. Cognitive performance (attention, declarative and working memory, executive functions and recognition of emotions) was assessed in 14 abstinent cocaine-dependent subjects in outpatient treatment and 13 control participants. Three salivary samples were collected at home by all the participants in the morning, afternoon and at bedtime. Patients showed higher levels of cortisol in the morning, as well as higher area under the curve with respect to the ground (AUCg). Regarding cognitive performance, cocaine-abstinent subjects showed worse performance in attention (*d*2 test), verbal memory (Spanish Complementary Verbal Learning Test, TAVEC) and executive tests (Tower of Hanoi and phonological fluency test) with respect to the control group. Morning cortisol levels and the AUCg index were negatively associated with the age of onset of drug consumption and the AUCg index was also positively associated with craving in our patients’ group. Moreover, morning cortisol levels, as well as the AUCg index, were negatively associated with verbal memory performance. Therefore, our pilot study suggests that salivary cortisol measurements could be a good avenue to predict craving level, as well as cognitive status, especially the declarative memory domain.

## 1. Introduction

Cocaine addiction is a mental relapsing disorder the underlying mechanisms of which are not well understood. Amongst all of them, activation of the hypothalamic–pituitary–adrenal (HPA) axis seem to be a prerequisite involved in cocaine abuse-related effects since adrenalectomized rats did not learn to self-administer cocaine [1,2], while corticosterone administration facilitated the acquisition of cocaine self-administration [3,4,5]. Moreover, it has also been observed that the central action of cocaine is supported by the enhancement of HPA hormones after intracerebroventricular (i.c.v.) administration [6,7]. Specifically, cocaine can stimulate the release of adrenocorticotropin hormone (ACTH) and cortisol/corticosterone in both humans and animal models in a dose-dependent way [8,9]. Therefore, it is undeniable that stress is a factor highly involved in cocaine addiction and the impact of this drug on the stress response seems to play an important role in the transition from the recreational or intermittent consumption to dependence [10,11]. Along with this, chronic abuse of drugs, such as cocaine, also induces a dysregulation of the HPA axis and extra-hypothalamic stress systems, which may explain the ability of stress to enhance the vulnerability to relapse [12,13,14,15]. Hence, abstinence from drugs of abuse can lead to increased anxiety, making addicts more vulnerable to the effects of stress [16,17,18,19]. For instance, acute administration of cortisol increased cocaine craving in individuals with cocaine use disorder compared to a placebo injection [20]. Moreover, increased cortisol levels have been also associated with withdrawal symptoms and early relapse [21,22,23]. On the other hand, we also consider that some persons can take drugs to cope with stress-related symptoms and situations. Moreover, individuals who suffer from mental disorders, such as anxiety or depression, abuse drugs at higher rates than the general population.

It is well known that high levels of cortisol (hypercortisolism) are often observed in several disorders characterized by the presence of cognitive deficits, such as in Cushing’s syndrome, major depression, or in some forms of dementia [24,25,26]. Furthermore, prolonged glucocorticoid therapy for inflammatory diseases is also associated with deficits in declarative memory and healthy adults also show a decreased cognitive function after exogenous cortisol administration [27,28,29,30,31]. Several hippocampus changes have been identified to explain, at least in part, these mnemonic deficits related to high cortisol levels, such as alterations in the motivation–arousal–emotion systems, modifications in long-term potentiation, structural and functional neuronal and glia changes, and suppression of adult neurogenesis [32,33]. Along the same lines, chronic cocaine abusers and abstinent subjects with increased plasma cortisol values also displayed hippocampal-dependent learning and memory impairments [34]. Specifically, this study was the first to suggest that learning and memory deficits in cocaine-dependent individuals were associated with enhanced salivary cortisol after inpatient treatment [34]. Nevertheless, some studies seem to suggest that abnormalities in diurnal ACTH and cortisol levels or in pituitary response to corticotropin-releasing hormone (CRH) that may develop during cocaine abuse appear to normalize during extended abstinence from cocaine [35].

Despite preclinical and clinical studies having reported difficulties in attentional processes, processing speed or executive functions after chronic cocaine consumption, to date there have been no studies that have explored the involvement of cortisol levels in these deficits during the early/intermediate abstinence (≤3 months) [36,37,38,39]. This is a remarkable point because the neuropsychological status constitutes one of the most consistent risk factors for treatment dropout observed in recent studies [40,41,42]. Taking this into account, our aim was to carry out a study to explore whether basal salivary cortisol in drug-abstinent individuals provides a reliable avenue to predict craving, which is a factor highly involved in the probability of relapse, as well as the cognitive performance involved in treatment adherence. Although our sample size is limited, we hypothesize that subjects with substance use disorder (SUD) will have higher cortisol levels which may be associated with poorer cognitive performance and higher craving. Finally, we have selected this type of sample collection because it has recently emerged as a reliable way of evaluating HPA function and it counts upon several advantages. For instance, its collection technique is non-invasive, inexpensive, easily obtainable and it has been shown to have a significant positive association with plasma cortisol [34,43,44].

## 2. Material and Methods

### 2.1. Participants

Male white Caucasian participants with SUD (*n* = 14) and a healthy control group (*n* = 13) constituted our sample. Individuals with SUD were recruited from outpatient detoxification treatment programs at Ayuda a la Recuperación de Enfermos Alcohólicos (AREA) and Fundación CESMA Proyecto Hombre, in Malaga, Spain and healthy subjects were recruited by convenience in the same city. SUD diagnosis was considered when the recurrent use of drugs causes clinically significant impairment, including health problems, disability, and failure to meet major responsibilities at work, school, or home. All participants were informed about the aim of the study and then they provided written consent to participate. The inclusion criteria were: (1) aged 25 to 55 years; (2) a diagnosis of SUD, with cocaine as the primary drug (cocaine use >1.5 g per month); (3) no consumption of prescription drugs; (4) abstinence duration between 2 and 8 weeks which was verified weekly by using the Multidrug urine test Instant View (Alfa Scientific Designs Inc., Gregg St, Poway, CA 92064, USA); (5) elementary/primary school completed; and (6) an absence of axis I diagnoses aside from alcohol abuse and/or nicotine dependence. Diagnoses of SUD were based on clinical and structured interviews following the DSM-5 criteria. For healthy controls, the inclusion criteria were the same including no history of drug abuse (counting nicotine and alcohol). Volunteers were excluded whether they presented severe difficulties in understanding the test instructions, altered consciousness or agitation, and if they consumed prescription drugs affecting the central nervous system. Moreover, volunteers taking medical treatments with possible effects on neuroendocrine measures were also excluded. Detailed information about the sample is displayed in Table 1.

The study and protocols were approved by the Ethics Committee of the University of Malaga (CEUMA: 67-2019-H) in accordance with the Ethical Principles for Medical Research Involving Human Subjects adopted in the Declaration of Helsinki by the World Medical Association (64th WMA General Assembly, Fortaleza, Brazil, October 2013), Recommendation No. R (97) 5 of the Committee of Ministers to Member States on the Protection of Medical Data (1997), and the Spanish Data Protection Act (Ley Orgánica 15/1999 de Protección de Datos, LOPD).

### 2.2. Procedures

All information gathered was encrypted to preserve privacy and confidentiality. Once informed consent was obtained, trained psychologists carried out a structured interview to collect data on sociodemographic variables and substance abuse (age of onset, drugs used, years of abuse, last use, etc.), along with a Likert scale of craving from 0 to 10 to determine the desire for consuming drugs during the current time, last week, and previous month (maximum 30 points). A cognitive reserve (CR) questionnaire was then conducted [45]. This scale comprises 8 items that evaluate schooling level (from 0 to 5); parents’ schooling level (from 0 to 2); formal courses taken (from 0 to 3); musical training (from 0 to 2); languages (from 0 to 3); reading activity (from 0 to 4); work achievements (from 0 to 4); and intellectual games practice (from 0 to 2). A high score on these variables would suggest a higher CR, with a maximum score of 25 points. We have chosen this questionnaire because it is both useful and quick to administer in clinical settings, based on the assessment of the most relevant variables related to the construct of CR [39]. Afterwards, participants underwent a comprehensive neuropsychological evaluation in a single session (approximately 2 h). Participants could revoke their consent at any time. Along with this, we received a medical report from each patient specifying whether they had any type of psychopathological disorder. Based on this clinical information, the absence of a characterized depression and the non-use of antidepressants was considered an exclusion criterion.

### 2.3. Neuropsychological Assessments

Attentional performance, psychomotor speed and visual searching were evaluated with the d2 test and the Trail Making Test, part A [46,47]. Verbal and non-verbal declarative memory were tested with the Spanish Complementary Verbal Learning Test (TAVEC) and the Rey–Osterrieth Complex Figure Test, respectively [48,49]. The copy part of the latter test allowed us to discard perceptive alterations. Executive functions were evaluated with a variety of tests, including the Tower Test [50], the Wechsler Memory Scale forward and backward digit amplitude task (WAIS-IV) [51], the Trail Making Test, part B [47], a verbal fluency test, and the Stroop Test [50,51,52]. Moreover, for emotional perception, we employed the Reading the Mind in the Eyes Test (RMET) [53]. All neuropsychological assessments were undertaken with pencil and paper. The mean ± standard error of mean (SEM) for each group in the individual tests is displayed in Table 2.

### 2.4. Salivary Collection Sample and Enzyme-Linked Immunosorbent Assay (ELISA)

At the end of the neuropsychological session, subjects were given 3 salivettes (Salivette^®^ Cortisol, Sarstedt, S.A., Barcelona, Spain) and collections were done at home in a single day. Patients were instructed on how to collect the samples between 08.00 to 09.00 before breakfast and at least one hour after waking to avoid interfering with the cortisol awakening response, at 16.00 to 17.00 and before going to sleep (23.00 to 24.00). Then, they had to store the saliva samples in the refrigerator until they were delivered to the laboratory where the samples were stored at −20°C. Once in the laboratory, the samples were centrifuged at 3000 rpm for 5 min, resulting in a clear supernatant of low viscosity that was stored at −80 °C until the analyses of the salivary cortisol levels. Salivary cortisol concentrations were measured using a commercially available enzyme-linked immunosorbent assay (ELISA, Salimetrics^®^, Carlsbad, CA 92008, USA) with intra- and inter-assay precision of 2.6 and 4.8%. Cortisol levels were expressed in μg/dL.

### 2.5. Statistical Analysis

All statistical analyses were performed using SPSS 25 (IBM SPSS Statistics, Corporate headquarters, New Orchard Road, Armonk, New York 10504-1722, USA) and all *p* values were two-tailed, and the level of significance was taken as *p* ≤ 0.05. Descriptive analyses were performed on the demographics, the information related to drug use (age of onset, years of substance use, weeks of abstinence, etc.) (Table 1) and the neuropsychological variables (Table 2). We reported mean ± standard error of the mean (SEM) and frequency (percentage) for continuous and categorical variables, respectively. Statistical differences between groups in cortisol levels (morning, afternoon and bedtime levels) were computed by one-way analysis of variance (ANOVA) with repeated measures. Honestly-significant-difference (HSD) Tukey’s post-hoc was performed when significant differences were found. We also calculated the area under the curve with respect to ground (AUCg) which reflects the total daily cortisol output [54,55]. The formula for calculation of the AUCg was: (((*morning cortisol + afternoon cortisol*) × *time interval between measurements*)/2) + (((*afternoon cortisol + bedtime cortisol*) × *time interval between measurements*)/2) [55]. Owing to the possible influence of the CR level on the cognitive performance, we carried out a multivariate analysis of variance (MANOVA) with CR as a covariable to analyze possible differences between groups in the neuropsychological tests controlling the effect of this variable [56].

Finally, Pearson correlation coefficients were performed for each group to analyze possible relationships between salivary cortisol measurements (morning levels and the AUCg) with those cognitive variables that also were different between groups. In the case of the SUD group, we also calculated the possible relationship between cortisol levels with drug-related variables. This analysis used Pearson’s *r* correlations ≥0.8 and *p* ≤ 0.005 (as previously described in Moreno-Fernandez et al., 2017; Sampedro-Piquero et al., 2018; Wheeler et al., 2013). Hence, this considered the probability to 0.005 is a reliable measure to avoid Type I errors in our study.

## 3. Results

### 3.1. Group Differences in Salivary Cortisol Levels

**Figure 1:**
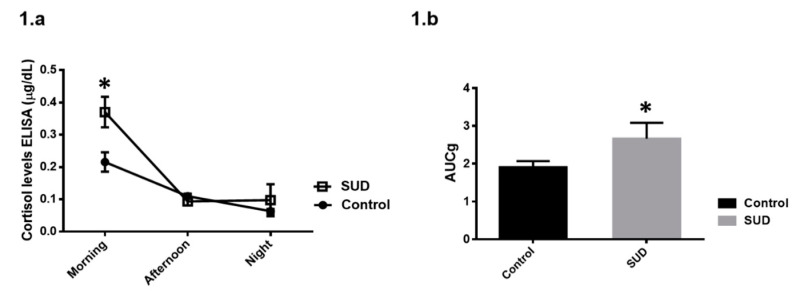
(**a**) Comparison between groups in basal salivary cortisol at three-day moments (μg/dL). As it can be appreciated, SUD group showed higher levels in the morning with respect to the control group (* *p* ≤ 0.05). (**b**) Area under the curve with respect to ground (AUCg) index reflecting an increase in the diurnal pattern of cortisol levels in the SUD group (* *p* ≤ 0.05).

On the one hand, one-way ANOVA with repeated measures revealed that cortisol levels were different over the day regardless of group condition (*F*(2, 50) = 50.50, *p* < 0.001). The factor *groups* was not significant (*F*(1, 25) = 3.54, *p* = 0.07), whereas the interaction *groups* × *cortisol levels* reached significance (*F*(2, 50) = 7.40, *p* = 0.002). HSD Tukey’s post hoc test indicated that the patients had higher cortisol levels in the morning compared to the control group (*p* = 0.002), whereas in the afternoon (*p* = 0.99) and bedtime (*p* = 0.99), we did not observe differences (Figure 1a). On the other hand, patients showed higher AUCg (*t*(25) = −2.27, *p* = 0.03) with respect to the healthy group (Figure 1b).

### 3.2. Group Differences in Cognitive Performance

Significant differences were found between groups in the CR level (*t*(25) = −2.38, *p* = 0.03), those being the patients who showed a lower level in this variable. Because of this, CR variable was used as a covariable when we compared both groups in the cognitive performance of the different neuropsychological tests.

#### 3.2.1. Attentional Processes and Psychomotor Speed

Patients showed a lower performance in the *d*2 test (TOT: *F*(1, 24) = 7.33, *p* = 0.01; CON: *F*(1, 24) = 6.08, *p* = 0.02; VAR: *F*(1, 24) = 4.36, *p* = 0.04), but significant differences were not found between groups in the time spent to complete the TMT part A and B (Table 2).

#### 3.2.2. Declarative Memory

Regarding the TAVEC test, patients recalled a lower number of words in the Trial 5 (*F*(1, 24) = 5.20, *p* = 0.03), showed an interference effect with list B (*F*(1, 24) = 6.11, *p* = 0.02), had a worse short-term and long-term memory recall than the CON group (short-term memory (STM): *F* (1, 24) = 6.75, *p* = 0.02; STM with cues: *F*(1, 24) = 6.34, *p* = 0.02; long-term memory (LTM): *F*(1, 24) = 9.61, *p* = 0.005; LTM with cues: *F*(1, 24) = 11.10, *p* = 0.003), and they used significantly less of a semantic strategy during the free LTM (*F*(1, 24) = 5.33, *p* = 0.03) and during the list acquisition (*F*(1, 24) = 4.90, *p* = 0.04). Moreover, patients committed a higher number of intrusions during free memory (*F*(1, 24) = 5.10, *p* = 0.03), as well as during the memory with semantic cues (*F*(1, 24) = 6.37, *p* = 0.02). In contrast, significant differences were not found in the copy and recall of the Rey Complex Figure (Table 2).

#### 3.2.3. Executive Functions and Emotional Perception

The SUD group performed worse than the CON group in the D-KEFS Tower test (*F*(1, 2) = 5.07, *p* = 0.03), and in the phonological verbal fluency test (*F*(1, 2) = 3.44, *p* = 0.05). In the rest of the executive measurements, significant differences were not observed, as well as in the execution of the RMET (Table 2).

### 3.3. Relationship between Salivary Cortisol Levels, Cognitive Status and Drug-Related Variables

#### 3.3.1. Control Group

Significant negative correlations were found between morning cortisol levels and the AUCg index with STM recall with semantic cues from the TAVEC test (*r* = −0.79, *p* = 0.004, *r* = −0.80, *p* = 0.004, respectively).

#### 3.3.2. SUD Group

Interestingly, we found a significant negative correlation between morning cortisol levels and the AUCg with the age of onset of consumption (*r* = −0.84, *p* = 0.003, *r* = −0.82, *p* = 0.004, respectively) and positive with craving levels (AUCg: *r* = 0.78, *p* = 0.005). Moreover, morning cortisol levels and the AUCg index were also positively associated with the number of intrusions during free memory (*r* = 0.87, *p* = 0.003, in both variables) and negatively with free STM (morning cortisol levels: *r* = −0.91, *p* = 0.002) and LTM (morning cortisol levels: *r* = −0.83, *p* = 0.003).

## 4. Discussion

This pilot experiment analyzed the relationship between salivary cortisol levels with both the cognitive status and drug-related variables. On the one hand, we found that patients had higher morning cortisol concentrations, as well as increased AUCg with respect to the healthy control group. We observed these cortisol measurements were positively associated with craving levels and negatively with the age of onset of drug consumption. Some studies about this topic have revealed that a deviation of cortisol secretion away from the homeostatic diurnal pattern in cocaine-dependent subjects was associated with reduced success at achieving early abstinence [23,24,25,26,27,28,29,30,31,32,33,34,35,36,37,38,39,40,41,42,43,44,45,46,47,48,49,50,51,52,53,54,55,56,57]. This finding suggests a feed-forward cascade of effects of drugs on stress biology [58]. Hence, chronic drug users are more vulnerable to negative affect, driving a cycle of increased drug use and stress disruption that further drive greater compulsive drug motivation and relapse risk. Additionally, activation of the HPA axis has been frequently found in cocaine abusers along with increased drug craving and subjective anxiety [18]. Likewise, this HPA activity enhancement seems to be also involved in cocaine reinforcement and relapse [12,13,14,15,59]. On the other hand, our sample was composed of early/intermediate abstinent individuals (between 2 and 8 weeks), so it is possible that the higher cortisol levels observed could be due to a negative affect state characterized by irritability, anxiety, emotional distress or craving which is common during this period [60]. Preclinical studies point in the same direction revealing that withdrawal from repeated administration of cocaine produces an anxiogenic-like response in the elevated plus maze and defensive burying test [61,62]. Furthermore, this association between cortisol and craving could be a predictor of early dropout, especially in early abstinence, when stress may be intensified by the hypodopaminergic condition resulting from drug withdrawal. Hence, interventions focused on regulating the stress response seem to be important elements in this initial stage of treatment. Regarding the association between morning cortisol levels and early age of onset, this could be related to a greater history of cocaine consumption [19]. Concerning this, some studies have demonstrated the influence of parental history of substance abuse on the risk of early drug abuse in their offspring [63,64], and for instance, higher morning cortisol levels have been found among crack cocaine users with positive family history and lower treatment retention [44]. Indeed, activation of the HPA response may be an early dysregulation associated with excessive and early drug taking that ultimately sensitizes the extrahypothalamic CRH systems [14,15,16,17,18,19,20,21,22,23,24,25,26,27,28,29,30,31,32,33,34,35,36,37,38,39,40,41,42,43,44,45,46,47,48,49,50,51,52,53,54,55,56,57,58,59,60,61,62,63,64,65].

Regarding cognitive performance, cocaine-abstinent subjects showed worse performance in attention (*d*2 test), verbal memory (TAVEC) and executive tests (Tower of Hanoi, phonological fluency test) with respect to the control group. According to this, several studies about the impact of cocaine on cognitive domains have described a broad neuropsychological impairment characterized by deficits in attentional and visuospatial domains, short-term and working memory, and executive performance involving abstract reasoning, cognitive flexibility, and inhibitory control [66,67,68,69]. Even more, recreational and non-dependent degree of cocaine consumption also led to cognitive deficits [70]. Nevertheless, although the main consumed drug was the cocaine, all the subjects consumed alcohol, so we cannot discard the possibility that the concomitant use of cocaine and alcohol may have additive negative effects on cognition as compared to the use of only one of these two substances [71]. Therefore, it seems necessary to determine the neuropsychological profile of subjects with SUD as they may be part of the set of factors that maintain drug use [42]. Additionally, these cognitive deficits may alter the awareness of the addiction problem, as well as the follow-up of the treatment program and the assimilation of instructions which demand a high cognitive challenge [41,72,73,74].

Owing to substance abuse seeming to induce cognitive deficits, as well as an altered HPA axis functioning, we aimed to study whether the cortisol measurements which showed significant differences between groups (morning cortisol levels and the AUCg), could be related to the performance in the different neuropsychological variables registered, as well as to drug-related variables. As a result, we observed that both morning cortisol levels and AUCg were associated with poorer memory recall. Despite the scarce evidence in subjects with SUD, other investigations carried out with an older population showed that higher cortisol levels were associated with poorer performance on memory, processing speed, and executive functioning [54,75,76]. In this regard, cortisol has effects on learning and memory, mainly influencing memory consolidation and retrieval processes [77,78,79,80]. Fox et al.’s group observed that learning and memory deficits in cocaine-dependent individuals are associated with enhanced morning cortisol and with cocaine use outcomes after inpatient treatment [34]. Our findings are consistent with recent addiction models suggesting that chronic cocaine-related neuroadaptations affect learning and memory function, which in turn influences drug-use outcomes. Exposure to elevated cortisol levels are involved in hippocampal alterations which could mediate in declarative memory dysfunction and hippocampal disinhibition [33,81]. Additionally, preclinical studies have suggested a role of the CRH_2_ receptor in mediating memory dysfunction and stress vulnerability associated with psychostimulant drug withdrawal [82]. We also observed that higher morning cortisol was associated with a higher number of intrusions in the TAVEC test. This measurement could be more related to the executive domain because it requires inhibiting incorrect responses following interference. This domain has been shown to be affected in patients with prolonged corticosteroid treatments [31,83,84].

As was previously mentioned, our patient sample showed an altered performance in selective and sustained attention. In this regard, cocaine abuse has been shown to be associated with poor performance in cancellation tests, as administered in our study [85]. In addition, cocaine has been recognized as a drug that induces reduced visual attention and an altered ability to control attentional focus compared to non-cocaine drug users [86]. Concerning to the effectiveness in the *d*2 test, we observed that the performance was negatively associated with morning cortisol levels. In general, the literature about this topic suggests a facilitator role for moderate levels of cortisol in studies on arousal, attention and concentration [31,87]. Nevertheless, apart from alterations in the HPA axis, we must consider other potential factors which could be related to the worse cognitive performance observed in the SUD group. For instance, variables such as low educational level, school dropout, low socioeconomic status, genetic aspects, certain cognitive traits, as well as the pattern of drug consumption (poly-drug abuse, high frequency and quantity, early age of onset, etc.) could also lead to the cognitive deficits observed in these subjects. Interestingly, we also observed that individuals with SUD performed equally well as controls in some of the cognitive tests, which suggests that they do not present a broad cognitive impairment, but rather one focused on certain cognitive areas.

Finally, this pilot experiment has several limitations that need to be considered and addressed in future studies. Regarding the sample, its size prevents the data from being generalizable and it would be also interesting to have collected data about treatment retention since recent studies have associated cortisol levels with this variable [44]. Moreover, although the main drug of consumption was cocaine, most of our subjects were poly-drug users, so we cannot totally rule out the effect of other substances, such as alcohol, on cognitive performance. Moreover, it would also be interesting to have collected salivary samples on 2–4 consecutive days to compare the results [88]. Moreover, although parameters such as menstrual cycle, sex hormones and gender seem to influence HPA axis and, in consequence, cortisol levels, it would have been also interesting to include a group of females in our study.

## 5. Conclusions

Despite this being a pilot study, our results suggest that dysregulation of stress responses may act as a potential predictor of craving and cognitive functioning, particularly in the field of declarative verbal memory. Hence, the identification of specific biomarkers associated with dysregulated responses to stress could allow us to identify new treatment strategies focused on the stress response improving the addiction prognosis. Nevertheless, our conclusions also suggest the need for further research to elucidate how substance abuse modulates the HPA axis and alters cognitive function.

## Figures and Tables

**Table 1:** Demographic, substance use characteristics of participants.

	SUD Group	Healthy Group
**DEMOGRAPHICS**		
Age ± SEM	36.2 ± 2.3	40.6 ± 3.2 (*p* = 0.27)
Sex % male	100%	100%
Years of education	15.7 ± 0.5	17.3 ± 0.8 (*p* = 0.10)
Marital status (single)—% (*n*)	64.3% (9/14)	38.46% (5/13)
**SUBSTANCE USE**		
Onset age	16.4 ± 0.8	
Duration of cocaine use (years)	18.7 ± 3.2	
Weeks since last used drug	5.9 ± 0.6	
Number of drugs consumed	3.1 ± 0.3	
Cocaine used per day (g)	1.5 ± 0.3	
Route of administration % intranasal	100%	
Reported tobacco use—% (*n*)	78.6% (11/14)	
Tobacco cigarettes per day	9.2 ± 1.9	
Reported alcohol use—% (*n*)	100%	
Reported cannabis use—% (*n*)	28.6% (4/14)	
Reported amphetamine/MDMA use—% (*n*)	0% (0/14)	
Score craving scale (maximum 30)	12.9 ± 1.5	

Values are means ± standard error of the mean (SEM) or percentages. Substance use disorder (SUD) group, *n* = 14, Control group, *n* = 13.

**Table 2:** Neuropsychological tests results.

	Healthy Controls	SUD Group	Student’s *t*-Test and *p* Values for Comparisons	Cohen’s d
**COGNITIVE RESERVE**	11.70 + 0.99	9.07 + 0.53	*t*(25) = −2.38, *p* = 0.03 *	0.9
**Attention, psychomotor speed and visual searching**				
**d2**				
-Effectiveness index	421.9 + 28	333.6 + 17.9	*F*(1,24) = −7.33, *p* = 0.01 *	1.03
-Concentration index	158.8 + 11.7	106.1 + 13.9	*F*(1,24) = 6.08, *p* = 0.02 *	1.10
-Variability index	12.8 + 1.3	19 + 2.1	*F*(1,24) = 4.36, *p* = 0.04 *	1.23
**Trail making test**				
-Version A (s)	24.9 + 2.3	32.1 + 5.2	*F*(1,24) = 0.12, *p* = 0.73	
-Version B (s)	64.9 + 7.4	83.5 + 13.4	*F*(1,24) = 0.25, *p* = 0.62	
**Declarative memory**				
**TAVEC**				
-Trial 1	7.2 + 0.4	6 + 0.5	*F*(1,24) = 1.79, *p* = 0.19	
-Trial 5	14.2 + 0.4	12.1 + 0.7	*F*(1,24) = 5.20, *p* = 0.03 *	1.3
-Total trials	58.6 + 1.5	48.3 + 2.8	*F*(1,24) = 6.29, *p* = 0.02 *	1.4
-Free STM	6.7 + 0.3	4.9 + 0.2	*F*(1,24) = 6.75, *p* = 0.02 *	1.2
-STM with semantic cues	13.8 + 0.7	10.6 + 0.8	*F*(1,24) = 6.34, *p* = 0.02 *	1.5
-Free LTM	14.2 + 0.4	10.5 + 0.8	*F*(1,24) = 9.61, *p* = 0.005 *	1.8
-LTM with semantic cues	14.4 + 0.4	10.6 + 0.8	*F*(1,24) = 11.10, *p* = 0.003 *	2.5
-Semantic strategy use during the list acquisition	18.8 + 2.8	10.9 + 2.1	*F*(1,24) = 4.90, *p* = 0.04 *	0.9
-Semantic strategy use during free STM	6 + 0.8	3.8 + 0.9	*F*(1,24) = 1.65, *p* = 0.21	
-Semantic strategy use during LTM	7.2 + 1	3.6 + 0.8	*F*(1,24) = 5.33, *p* = 0.03 *	1.6
-Serial strategy use during the list acquisition	5 + 1.2	5 + 0.9	*F*(1,24) = 0.02, *p* = 0.90	
-Serial strategy use during free STM	1 + 0.4	0.8 + 0.3	*F*(1,24) = 0.18, *p* = 0.68	
-Serial strategy use during free LTM	0.8 + 0.5	0.9 + 0.4	*F*(1,24) = 0.001, *p* = 0.97	
-Intrusions during free memory	2.2 + 0.8	6.8 + 1.3	*F*(1,24) = 5.10, *p* = 0.03 *	1.3
-Intrusions during memory with semantic cues	0.8 + 0.3	3.2 + 0.7	*F*(1,24) = 6.37, *p* = 0.02 *	2.1
-Recognition	15.8 + 0.1	15.7 + 0.2	*F*(1,24) = 0.04, *p* = 0.84	
-False positives in Recognition	0.7 + 0.2	2.8 + 1.1	*F*(1,24) = 1.27, *p* = 0.27	
**Rey Complex Figure**				
-Copy score	35.7 + 0.3	33.9 + 1.7	*F*(1,24) = 0.55, *p* = 0.47	
-Delayed score	23.8 + 1.6	20.3 + 1.8	*F*(1,24) = 2.27, *p* = 0.15	
**Executive functions**				
**D-KEFS Tower test**				
-Achievement score	20.2 + 0.8	16.7 + 0.8	*F*(1,24) = 5.07, *p* = 0.03 *	1.6
**Digit span**				
-Forward	5.9 + 0.3	5.8 + 0.3	*F*(1,24) = 0.28, *p* = 0.60	
-Backward	5.2 + 0.4	4.1 + 0.2	*F*(1,24) = 3.60, *p* = 0.06	
**Verbal fluency**				
-FAS	43.6 + 1.7	35.8 + 2.7	*F*(1,24) = 3.44, *p* = 0.05 *	
-Semantic: Animals	22 + 0.8	19.4 + 1.2	*F*(1,24) = 1.42, *p* = 0.24	0.9
**Stroop test**				
-Word	93.8 + 7	91.8 + 5.9	*F*(1,24) = 0.03, *p* = 0.85	
-Color	62.8 + 3.5	59.4 + 3.3	*F*(1,24) = 0.70, *p* = 0.41	
-Total Color/Word	49.4 + 6.8	36.9 + 2.3	*F*(1,24) = 2.49, *p* = 0.12	
-Interference Index	12.8 + 6	1.3 + 2.1	*F*(1,24) = 2.62, *p* = 0.12	
**Emotional perception**				
**RMET**				
-Correct answers	22.7 + 1.1	20.3 + 1.1	*F*(1,24) = 0.37, *p* = 0.55	

Values are means ± SEM or percentages. STM: Short-term memory; LTM: Long-term memory. *: Asterisks represent significant differences between groups (*p* ≤ 0.05).
